# Comprehensive Metabolomics Study in Children With Graves’ Disease

**DOI:** 10.3389/fendo.2021.752496

**Published:** 2021-11-16

**Authors:** Qin Xia, Weifeng Qian, Linqi Chen, Xiuli Chen, Rongrong Xie, Dandan Zhang, Haiying Wu, Hui Sun, Fengyun Wang, Jingjing Liu, Ting Chen

**Affiliations:** ^1^ Department of Endocrinology, Genetics and Metabolism, Children’s Hospital of Soochow University, Suzhou, China; ^2^ Department of Thyroid and Breast Surgery, The Affiliated Suzhou Hospital of Nanjing Medical University, Suzhou, China; ^3^ Department of Biochemistry and Molecular Biology, School of Medical and Biological Sciences, Soochow University, Suzhou, China

**Keywords:** Graves’ disease, children, serum metabolomics, untargeted metabolomics, metabolic pathway

## Abstract

**Objective:**

Graves’ disease (GD) related hyperthyroidism (HT) has profound effects on metabolic activity and metabolism of macromolecules affecting energy homeostasis. In this study, we aimed to get a comprehensive understanding of the metabolic changes and their clinical relevance in GD children.

**Methods:**

We investigated serum substances from 30 newly diagnosed GD children and 30 age- and gender-matched healthy controls. We explored the metabolomics using ultra-high-performance liquid chromatography–quadrupole time-of-flight mass spectrometry (UHPLC-QTOF/MS) analysis, and then analyzed the metabolomic data *via* multivariate statistical analysis.

**Results:**

By untargeted metabolomic analysis, a total of 730 metabolites were identified in all participants, among which 48 differential metabolites between GD and control groups were filtered out, including amino acids, dipeptides, lipids, purines, etc. Among these metabolites, 33 were detected with higher levels, while 15 with lower levels in GD group compared to controls. Pathway analysis showed that HT had a significant impact on aminoacyl-transfer ribonucleic acid (tRNA) biosynthesis, several amino acids metabolism, purine metabolism, and pyrimidine metabolism.

**Conclusion:**

In this study, *via* untargeted metabolomics analysis, significant variations of serum metabolomic patterns were detected in GD children.

## Introduction

Graves’ disease (GD) is the most common cause of hyperthyroidism (HT) with an autoimmune origin in children and adults (1–3). The incidence of GD in children is about 0.9-14.1/100,000, peaking in adolescent females (4–6). A trend of surging incidence of juvenile thyrotoxicosis was observed worldwide, with two to three times higher incidence in the Chinese pediatric population compared to the Caucasians (4–6).

It is well known that thyroid hormone regulates metabolic processes essential for normal growth and development in children (7). With excessive thyroid hormone, HT facilitates the metabolism process *via* elevated resting energy expenditure, weight loss, upregulated lipolysis, and gluconeogenesis, as well as decreased cholesterol levels (8).

Metabolomics is the study of specific small molecule metabolites or their profiles. Untargeted metabolomics provides a global fingerprint of information through the simultaneous identification of as many metabolites as possible within a tissue, biological fluid, or even cell sample (9, 10). Different instrumental platforms, including nuclear magnetic resonance (NMR) spectroscopy, gas chromatography (GC), and liquid chromatography (LC) coupled with mass spectrometry (MS), are used to cover different features of the human metabolome (9, 10). Metabolomics has been used to explore new biomarkers of disease risk in large-scale studies. In smaller studies, metabolomics has been designed to investigate the underlying mechanisms and progression of certain diseases, or to reveal the potential roles of dietary and environmental exposures, as well as gut flora activity in chronic diseases (11, 12). Multiple studies on metabolomic changes in GD adult patients have been reported, which showed that acylcarnitine and polyamine profiles were different between GD patients and healthy controls (13, 14). Metabolic pathways, such as arginine and proline metabolism and aminoacyl-transfer ribonucleic acid (tRNA) biosynthesis have also been altered in GD patients (15). However, the metabolomic alterations of GD in pediatric population have not been fully explored.

In the present study, we used an ultra-high-performance liquid chromatography coupled with the quadruple time-of-fight mass spectrometry (UHPLC-QTOF/MS)-based untargeted metabolomics approach to explore the perturbation of metabolic process in GD children compared to age- and gender- matched healthy normal controls. We aim to extend the current knowledge beyond previously reported targeted metabolite changes by examining the global serum metabolomics profiles of GD, and propose new dietary suggestions which may improve the treatment of GD.

## Materials and Methods

### Study Design and Participants

Blood samples of 30 newly diagnosed drug-naïve GD patients at Children’s Hospital of Soochow University from March 2017 to May 2018 were collected for our study. Meanwhile, 30 age- and sex-matched healthy controls were enrolled from their annual physical examination. GD was diagnosed according to guidelines (16) and as previously described (17).

All patients and control subjects underwent general physical examination and laboratory evaluation before enrollment. We excluded subjects with liver dysfunction, cardiovascular complications, or other endocrine disorders and immune diseases. Moreover, to avoid the impact of sex hormones on metabolites, we only included prepubertal children in this study, which means only boys and girls at Tanner stage I were recruited in the study.

### Sample Collection

Serum levels of thyroid-related hormones, including total thyroxine (TT4), total triiodothyronine (TT3), free thyroxine (FT4), free triiodothyronine (FT3), and thyroid stimulating hormone (TSH), as well as thyroid autoantibodies, containing thyroid peroxidase antibody (TPOAb), thyroglobulin antibody (TGAb), and thyroid stimulating hormone receptor antibodies (TRAb) were measured by electrochemiluminescence immunoassay at the laboratories of our hospital. Serum samples for untargeted metabolomics were taken after 10-12h night fasting from an antecubital venous catheter. Samples were placed on ice, separated within 20min, and stored at -80°C until analysis.

### Untargeted Metabolomics Analyzed by UHPLC-QTOF/MS

Samples were thawed at 4°C on ice. Then a 100μL sample was extracted by adding 400μL of extraction solvent (V methanol: V acetonitrile= 1:1, containing internal standard 2 μg/mL), vortexing for 30s, sonicating for 10min at 4°C, and then incubating for 1h at -20°C. The precipitated protein was then centrifuged at 4°C and 12000rpm for 15 min. Subsequently, the 425μL supernatant was dried in a vacuum concentrator without heating, resolved by 100μL extraction solvent (V acetonitrile: V water= 1:1), vortexed for 30s, sonicated for 10min at 4°C, and centrifuged for 15min at 12000rpm, 4°C. Then the supernatant (60μL) was transferred into a LC/MS vial for UHPLC-QTOF/MS analysis. To ensure data quality, 10μL supernatant from different individual serum samples were pooled as a quality control sample.

Metabolomics performed were described in a previous study (14). In brief, LC-MS/MS analyses were performed using a 1290 UHPLC system (Agilent Technologies, Santa Clara, CA, USA) with a UPLC BEH Amide column (1.7μm, 2.1*100mm, Waters) coupled to Triple time-of-flight 6600 (Q-TOF, AB Sciex, Framingham, MA, USA). The injection volume for each sample was 1μL. The mass spectroscopy (MS) data were collected from m/z 50-1200 Da. The MS spectra acquisition was performed using Analyst TF 1.7 software (AB Sciex) based on the information-dependent basis (IDA) mode. In each cycle, 12 precursor ions whose intensity greater than 100 were chosen for fragmentation at collision energy (CE) of 30 eV (15 MS/MS events per 50 ms of product ion accumulation time). The electrospray ionization (ESI) source conditions were set as following: nebulizer pressure, 60 Psi; auxiliary pressure, 60 Psi; curtain gas, 35 Psi; source temperature 650°C; ion spray voltage floating (ISVF) 5000 V or - 4000 V in positive or negative modes, respectively.

### Statistical Analysis

The UHPLC-QTOF/MS data analysis was performed as previously described (18).Briefly, MS raw data (.wiff) files were converted to the mzXML format using Proteo Wizard, and processed by R package XCMS (version 3.2). The preprocessing results generated a data matrix that consisted of the retention time (RT), massto-charge ratio (m/z) values, and peak intensity. R package CAMERA was used for peak annotation after XCMS data processing. In-house MS2 database was applied in metabolite identification. The SIMCA 14.1 software package (Unetrics, Umea, Sweden) was used to analyze the metabolites. Both principal component analysis (PCA) and orthogonal partial least squared-discriminant analysis (OPLS-DA) were used for the multivariate data analysis (MVDA). The SPSS 25.0 software (SPSS Inc., Chicago, IL, USA) was used to determine significant differences between GD and normal control groups. The metabolic features with both variable importance in projection (VIP) value > 1.5 and fold change (FC) >1.2 or <0.83 in the OPLA-DA model and values *P*<0.05 were considered significantly different. The correlations between substances and thyroid function, as well as autoantibodies, were analyzed *via* Spearman rank correlation and *P*<0.05 was considered as statistically significant.

When comparing quantitative variables between 2 groups, for normally distributed data, Student’s t-test was used and the results were expressed as means ± standard deviations. For data not normally distributed, Mann-Whitney *U*-test was used, and the results were expressed as medians (25th-75th percentiles).

## Results

### Demographics of the Study Population

The baseline clinical and biochemical characteristics of 30 GD patients and 30 age- and gender-matched healthy controls were shown in **Table 1**. As expected, girls (83.3%) are more susceptible to GD than boys (16.7%). Although all in normal ranges, GD patients had higher alanine aminotransferase (ALT), aspartate aminotranspherase (AST), gamma-glutamyl transpeptidase (GGT), alkaline phosphatase (ALP), direct bilirubin (DBIL), triglyceride (TG), and lower total cholesterol (TCHOL) levels than normal controls.

**Table 1:** Clinical and biochemical characteristics of the GD and control groups.

	Normal range	GD group (n = 30)	Control group (n = 30)	*p* Value
Age (months)^a^	NA	78.80 ± 20.50	72 ± 20.42	NS
Girls/Boys	NA	25/5	25/5	—
Wt (Kg)^b^	NA	24.86 (20.38~28.25)	26.48 (22.73~29.16)	NS
FT3 (pg/ml)^a^	2.71-4.69	11.41 ± 6.0	3.91 ± 0.32	<0.01
FT4 (ng/dl)^a^	1.04-1.83	4.33 ± 2.41	1.37 ± 0.16	<0.01
TSH(μIU/ml)^a^	0.91-4.63	0.0067 ± 0.0039	2.61 ± 1.31	<0.01
TT3 (ng/ml)^a^	0.81-2.43	3.18 ± 1.45	1.12 ± 0.23	<0.01
TT4 (ng/ml)^a^	55.33-124.22	157.92 ± 71.58	80.12 ± 13.33	<0.01
TPOAb (IU/ml)^a^	0.00-60.00	172.19 ± 39.16	44.57 ± 9.01	<0.01
TGAb (IU/ml)^a^	0.00-60.00	760.45 ± 1125.21	23.85 ± 9.72	<0.01
TRAb (IU/L)^a^	0.00-1.50	20.11 ± 11.06	0.51 ± 0.24	<0.01
ALT (U/L)^b^	5-35	26.70 (20.30~35.78)	12.70 (11.45~14.40)	<0.01
AST (U/L)^b^	10-67	28.50 (23.10~33.48)	24.50 (21.85~28.25)	<0.05
GGT (U/L)^b^	7-32	20.95 (14.10~33.73)	10.40 (8.80~11.60)	<0.01
ALP (U/L)^b^	0-500	295 (253~333)	209 (180~234)	<0.01
TBIL (μmol/l)^b^	3.40-17.10	8.80 (6.78~13.58)	8.40 (6.40~11.65)	NS
DBIL (μmol/l)^b^	0.00-10.00	3.61 (2.63~5.28)	2.70 (2.40~3.59)	<0.05
IBIL (μmol/l)^b^	0.00-17.00	5.15 (4.02~7.70)	5.90 (3.95~8.44)	NS
TP (g/l)^a^	60.0-83.0	66.03 ± 4.55	70.17 ± 4.02	NS
TG (mmol/l)^b^	0.00-1.70	0.83 (0.58~1.23)	0.65 (0.49~0.85)	<0.05
TCHOL (mmol/l)^a^	0.00-5.20	3.17 ± 0.52	4.54 ± 0.89	<0.05

GD, Graves’ disease; Wt, weight; FT3, free triiodothyronine; FT4, free thyroxine; TSH, thyroid stimulating hormone; TT3, total triiodothyronine;TT4, total thyroxine; TPOAb, thyroid peroxidase antibody; TGAb, thyroglobulin antibody; TRAb,thyroid stimulating hormone receptor antibodies; ALT, alanine aminotransferase; AST, aspartate aminotransferase; GGT, gamma-glutamyl transpeptidase; ALP, alkaline phosphatase; TBIL, total bilirubin; DBIL, direct bilirubin; IBIL, indirect bilirubin; TP, total protein; Tg, thyroglobulin; TCHOL, total cholesterol.

a The data were normally distributed.

b The data were not normally distributed. NA, Not available; NS, No significance.

### Differential Metabolites Between the GD and Control Groups

A total of 730 metabolites were identified in all participants. PCA score plots showed clustering of the control and HT groups with the cumulative fitness (R2 value) of the PCA model being 0.52 and 0.53, respectively, for positive and negative ion models (**Supplemental Figure 1**). The OPLS-DA analysis indicated clear separations between the HT and control groups both in positive (R2X=0.178, R2Y=0.895, Q2 = 0.727) and negative (R2X=0.152, R2Y=0.86, Q2 = 0.669) ion models (**Supplemental Figure 2**).

Based on the selection criteria including VIP > 1.5, *P* < 0.05, and FC > 1.2 or FC < 0.83, a total of 48 differential metabolites between GD and control groups were filtered out. Among these metabolites, 33 were detected with higher levels, while 15 with lower levels in GD group compared to controls (**Table 2**).

**Table 2:** Significantly changed metabolites of GD children compared to controls.

	Metabolites	VIP	P	FC
Amino acid metabolism	Isovalerylglycine	2.08	0	1.65
	L-Pipecolic acid	1.63	0	1.52
	L-Tyrosine	1.76	0	1.4
	L-Tryptophan	2.23	0	1.4
	L-Methionine	2.28	0	1.29
	L-Phenylalanine	2.48	0	1.3
	L-Threonine	1.8	0	1.28
	L-Glutamate	1.51	0	1.26
	Tyramine	2.46	0	1.24
	Creatinine	2.61	0	0.76
	Indoxyl sulfate	1.73	0.01	0.61
	Guanidinosuccinic acid	2.66	0	0.59
Dipeptides	gamma-L-Glutamyl-L-valine	2.8	0	1.73
	Gly-Glu	2.77	0	1.54
	Val-Met	3.08	0	1.48
	Ile-Ala	1.87	0	1.47
	Met-Tyr	1.61	0	1.45
	Pro-Glu	2.61	0	1.39
	His-Glu	1.72	0	1.35
	Phe-Glu	1.63	0.04	1.29
	Trp-Ile	1.97	0	1.22
	Ile-Val	2.41	0	0.57
Lipid metabolism	pregnenolone sulfate	2.21	0	2.71
	1-Palmitoyl Lysophosphatidic Acid	2.66	0	2.38
	Decanoyl-L-carnitine	2.13	0	1.87
	5-Oxo-ETE	1.57	0.01	1.45
	7-Oxocholesterol	1.73	0.02	1.44
	D-erythro-Sphingosine-1-phosphate	2.26	0	1.32
	Cortisone	1.32	0.03	1.22
	1-Palmitoyllysophosphatidylcholine	1.6	0.01	0.82
	Pristanic acid	1.58	0.01	0.81
	all cis-(6,9,12)-Linolenic acid	1.69	0.01	0.73
	Linoleic acid	2.02	0	0.73
	Pentadecanoic Acid	1.8	0	0.73
	Tridecanoic acid (Tridecylic acid)	1.55	0.02	0.72
	Sphinganine	2.22	0	0.69
	Myristic acid	2.12	0	0.64
	3b-Hydroxy-5-cholenoic acid	1.58	0.04	0.56
Tricarboxylic acid cycle	Isocitrate	1.66	0	1.77
	Succinate	1.59	0	1.47
Nucleotide metabolism	Hypoxanthine	1.56	0	1.41
	Xanthine	2	0	1.34
	S-Methyl-5’-thioadenosine	1.96	0	1.35
	5,2’-O-dimethyluridine	2.09	0	1.34
	2’-Deoxycytidine 5’-monophosphate (dCMP)	2.31	0	1.27
	5,10-methylene-THF	1.7	0.04	1.25
Others	Phenylacetic acid	1.51	0.04	0.8
	Protoporphyrin IX	1.89	0	0.55

### Differential Pathways Between the GD and Control Groups

**Figure 1:**
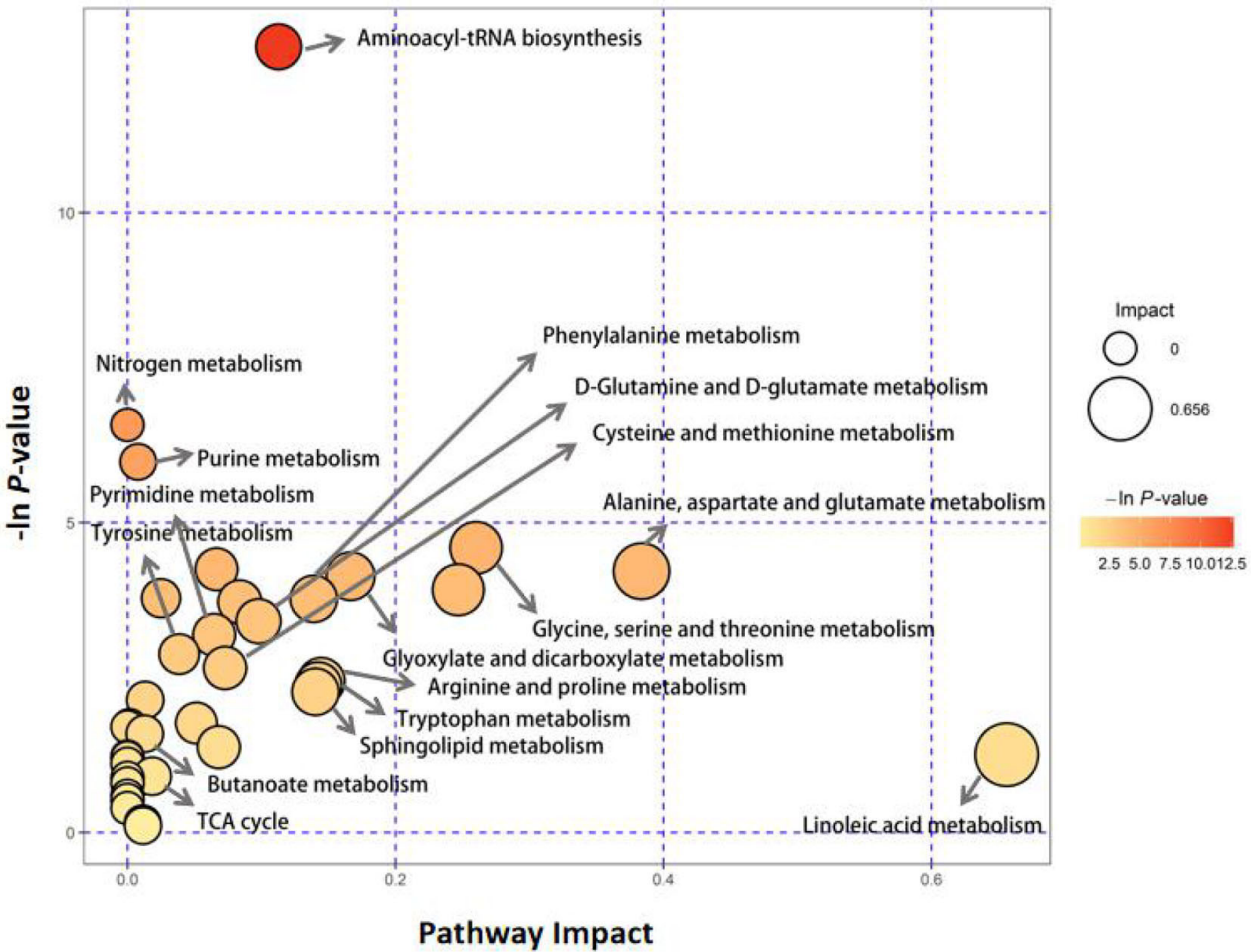
Pathway analysis of serum metabolite profiles of the hyperthyroidism group compared to the control group.

By comparing with the KEGG PATHWAY database (https://www.genome.jp/kegg/), the differentially abundant metabolites were cross-referenced with the related pathways. After enrichment and topology analysis, the impact values of each pathway were obtained. Essential pathways with large impacts were labeled in each comparison, with the detailed results of pathway analyses listed in **Figure 1** and **Table 3**. In GD group, the most significant changes were found in aminoacyl-tRNA biosynthesis, nitrogen metabolism, purine metabolism, alanine, aspartate and glutamate metabolism and phenylalanine metabolism.

**Table 3:** Differential pathways between the GD and control groups.

Pathway	Hits	-ln(p)	FDR	Impact
Aminoacyl-tRNA biosynthesis	10	12.68	0.00	0.11
Nitrogen metabolism	5	6.58	0.06	0.00
Purine metabolism	6	4.25	0.18	0.07
Alanine, aspartate and glutamate metabolism	3	4.22	0.18	0.38
Phenylalanine metabolism	4	4.13	0.18	0.17
Glycine, serine and threonine metabolism	4	3.92	0.18	0.25
Glyoxylate and dicarboxylate metabolism	4	3.78	0.18	0.02
D-Glutamine and D-glutamate metabolism	2	3.77	0.18	0.14
Tyrosine metabolism	5	3.72	0.18	0.08
Cysteine and methionine metabolism	4	3.41	0.22	0.10
Pyrimidine metabolism	4	3.19	0.25	0.06
Butanoate metabolism	3	2.88	0.32	0.04
Citrate cycle (TCA cycle)	2	2.66	0.37	0.07
Arginine and proline metabolism	4	2.44	0.44	0.14
Tryptophan metabolism	4	2.37	0.44	0.14
Sphingolipid metabolism	2	2.27	0.46	0.14
Linoleic acid metabolism	1	1.26	0.89	0.66

FDR, P value adjusted by false discovery rate; Hits, the matched number of metabolites in a pathway; Impact value, value calculated from pathway topography analysis.

### Relationship Between Thyroid Indices and Metabolites in GD Children

The correlations between thyroid indices and differential metabolite levels in GD patients were analyzed *via* Spearman’s correlation analysis. Correlations with Spearman rank correlation coefficient > 0.4 and *P* < 0.05 were filtered out and listed in **Table 4**. We found that three metabolites were associated with TT3, four with TT4, two with FT3, and three with FT4. Among the differential metabolites, three were associated with TPOAb, one with TRAb, two with TGAb, and one with TSH.

**Table 4:** Relationship between thyroid indices and metabolites levels in GD children.

Metabolites	Thyroid indices	Spearman rank correlation coefficient	*P* Value
L-Tryptophan	TGAb (IU/ml)	-0.770	0.009
L-Glutamate	FT4 (ng/dl)	0.424	0.027
S-Methyl-5’-thioadenosine	TSH (uIU/ml)	-0.464	0.046
S-Methyl-5’-thioadenosine	TPOAb (IU/ml)	-0.720	0.006
Guanidinosuccinic acid	TPOAb (IU/ml)	-0.687	0.01
Creatinine	TT4 (ng/ml)	-0.576	0.008
Creatinine	TT3 (ng/ml)	-0.596	0.006
Succinate	TPOAb (IU/ml)	-0.593	0.033
Succinate	TT3 (ng/ml)	-0.449	0.047
Succinate	FT4 (ng/dl)	0.434	0.024
D-erythro-Sphingosine-1-phosphate	TT4 (ng/ml)	0.544	0.013
Decanoyl-L-carnitine	TT4 (ng/ml)	0.484	0.031
Acetylvalerenolic acid	FT3 (pg/ml)	-0.578	0.015
3b-Hydroxy-5-cholenoic acid	FT3 (pg/ml)	0.502	0.04
1-Palmitoyl Lysophosphatidic Acid	FT4 (ng/dl)	0.522	0.005
Phe-Glu	TRAb (IU/L)	0.405	0.049
Met-Tyr	TGAb (IU/ml)	0.721	0.019
Indoxyl sulfate	TT3 (ng/ml)	-0.547	0.013
Indoxyl sulfate	TT4 (ng/ml)	-0.469	0.037

## Discussion

The present study showed that serum metabolic patterns were significantly different between GD children and healthy controls. A total of 730 metabolites were identified in all participants, among which 48 differential metabolites between GD and control groups were filtered out. To our knowledge, this is the first study analyzing the metabolic changes in GD children *via* an untargeted metabolomic method.

Amino acids are organic compounds that contain amine (-NH2) and carboxyl (-COOH) functional groups, along with a side chain (R group) specific to each amino acid (19). Amino acids are the monomers that make up human proteins and the second largest component of human muscle and other tissues. Besides, amino acids also actively participate in numerous biological processes, including biosynthesis and neurotransmitter transport (19). In the present study, we found that GD children had more active protein digestion/absorption and nitrogen metabolism. Quite a few amino acids, mostly essential amino acids, had much higher levels in GD children compared with controls. Interestingly, the amino acid pattern changes in GD children are quite different from those of adult patients. For example, significant changes of arginine and proline metabolism pathways were observed in GD adult patients (20), but not the children. This may be explained by the different amino acid metabolism among various age groups (20). Additionally, significantly elevated levels of phenylalanine and tyrosine were observed in GD children, but not in GD adults (20). Most phenylalanine is converted to tyrosine, a key component of thyroxine, through phenylalanine hydroxylase (21). Thus, reduction of phenylalanine intake in GD children, but not adults, may be a potential way to decrease their thyroxine production.

Tryptophan (Trp) is an essential amino acid that serves several important purposes and a precursor of the neurotransmitter serotonin. The tri-iodothyronine (T3) and thyroxine (T4) entry into different cell types depends on the aromatic amino acid transport system T, and there is a counter transportation between T3 and Trp. Thus, Trp supplementation may reduce the T3 uptake of cells (22, 23). Normal Trp metabolism has two main branches: 3-10% of Trp keeps the indole ring intact while producing chemical messengers such as serotonin, while the majority (90%) breaks the indole ring generating the kynurenine path, kynurenine, nicotinic acid, and the nicotinamide adenine dinucleotide (NAD^+^) (24). Previous studies have reported that thyroxine elevated the conversion ratio of Trp to nicotinamide, not through the kynurenine pathway but *via* aminocarboxymuconate semialdehyde decarboxylase (25). Our results were consistent with previous studies since no elevation of substrates in the kynurenine pathway was observed. Moreover, elevated 5-hydroy-indoleacetate were detected in the serum of GD children, indicating an intensified conversion of Trp to serotonin. Similar findings have been reported in the brain of adult GD patients, and the elevation of serotonin may contribute to the mood change of GD patients (26). Besides, we found that Trp levels in GD children were negatively correlated with TGAb levels, which may suggest a regulation role of Trp in the autoimmune process of GD. Indeed, previous studies have points to Trp degradation as a potent immunosuppressive mechanism involved in the maintenance of immunological tolerance. Therefore, Trp metabolism is quite important in the pathogenesis of GD, further studies are needed to explore the impact of supplementation and/or deprivation of Trp on GD pathogenesis.

Sphingosine 1-phosphate (S1P), a sphingolipid mediator, regulates various cellular functions *via* high-affinity G protein-coupled receptors, S1P1-5, and plays an important regulatory role in congenital and adaptive immune responses (27). Sphk1/S1P/S1PR signaling pathway can be used as a target for the treatment of autoimmune diseases (28). For example, the immunosuppressant Fingolimod (FTY720), a sphingosine analogue, is used as an S1PR antagonist in the treatment of multiple sclerosis (29). In addition, S1P lyase inhibitors can alleviate joint inflammation and destruction in rheumatoid arthritis mice (30). In recent years, Cheng Han et al. found that Sphk1/S1P/S1PR1 signal transduction is involved in the development of mice autoimmune thyroiditis (AIT). In AIT mice, the proportions of inflammation-related cell subtypes (such as Th1, Th17 and Tfh) are elevated, while FTY720 administration can decrease the their levels, suggesting that suppression of Sphk1/S1P/S1PR1 signaling pathway may be a potential therapeutic target of AIT (31). In this study, we found that sphinganine decreased, while S1P levels increased in the GD group. Besides, S1P levels positively correlate with TT4 levels. Thus, we suppose that FTY720 is also promising in the treatment of GD.

We detected much higher levels of pregnenolone sulfate in GD patients, which is consistent with previous studies (32). Pregnenolone sulfate is the source of steroid synthesis pathway. More importantly, pregnenolone sulfate regulates the release of multiple neurotransmitters and is crucial to multiple brain functions (33). The elevated pregnenolone sulfate levels may exert important effects on the neurodevelopment of GD children, but further studies are still needed to elucidate the consequences.

Protoporphyrin IX levels were significantly lower in GD children compared to those of controls. This can be easily explained by the fact that protoporphyrin IX is a crucial constituent of thyroid peroxidase (TPO), and more protoporphyrin IX is transported into the thyroid gland in GD patients. Interestingly, exacerbation of erythropoietic protoporphyria and acute intermittent porphyria has been reported in a few patients with HT patients (34, 35). Since these situations are quite rare and a decrease of protoporphyrin IX is observed in otherwise normal GD children, the correlation of HT and porphyria needs further investigation.

Our study has several limitations. Firstly, the sample size was relatively small, and further studies with larger sample size are needed to confirm our findings. Secondly, the main findings need to be validated by a second method. Thirdly, because this study included only local Chinese children, our results may not apply to other populations.

In conclusion, GD children have highly different serum metabolomic patterns compared to healthy controls, which may be induced either by HT or by autoimmunity. Our study is the first study addressing metabolic changes in GD children *via* untargeted metabolomic analysis. More importantly, our study provides comprehensive insights into the changes of metabolic processes, which is beneficial in improving the understanding and treatment of GD children.

## Data Availability Statement

The original contributions presented in the study are included in the article/**Supplementary Material**. Further inquiries can be directed to the corresponding author.

## Ethics Statement

The studies involving human participants were reviewed and approved by Ethics Committee of Children’s Hospital affiliated to Suzhou University. Written informed consent to participate in this study was provided by the participants’ legal guardian/next of kin.

## Author Contributions

Conceived and designed the experiments: TC; Performed the experiments: QX, WQ, LC, XC, RX, DZ, HW, HS, FW, JL; Analyzed the data: QX, WQ and TC; Wrote the paper: TC, QX, and WQ. All authors contributed to the article and approved the submitted version.

## Funding

This study was supported by Suzhou Personnel Planning Project (project code GSWS2019051 and GSWS2020046), and Suzhou Science and Technology Development Project (SS22064) awarded to TC; a National Natural Science Foundation of China (project code 31701251) awarded to JL. This study was also supported by the Department of Pediatrics Clinical Center of Suzhou (Szzx201504).

## Conflict of Interest

The authors declare that the research was conducted in the absence of any commercial or financial relationships that could be construed as a potential conflict of interest.

## Publisher’s Note

All claims expressed in this article are solely those of the authors and do not necessarily represent those of their affiliated organizations, or those of the publisher, the editors and the reviewers. Any product that may be evaluated in this article, or claim that may be made by its manufacturer, is not guaranteed or endorsed by the publisher.
