# Supplementary material for: Comprehensive Metabolomics Study in Children With Graves’ Disease
Source: Front Endocrinol (Lausanne). 2021 Nov 16;12:752496. doi: 10.3389/fendo.2021.752496 (PMC8635134; doi:10.3389/fendo.2021.752496)

Supplemental Figure 1. PCA score plot of the control, and hyperthyroidism groups.

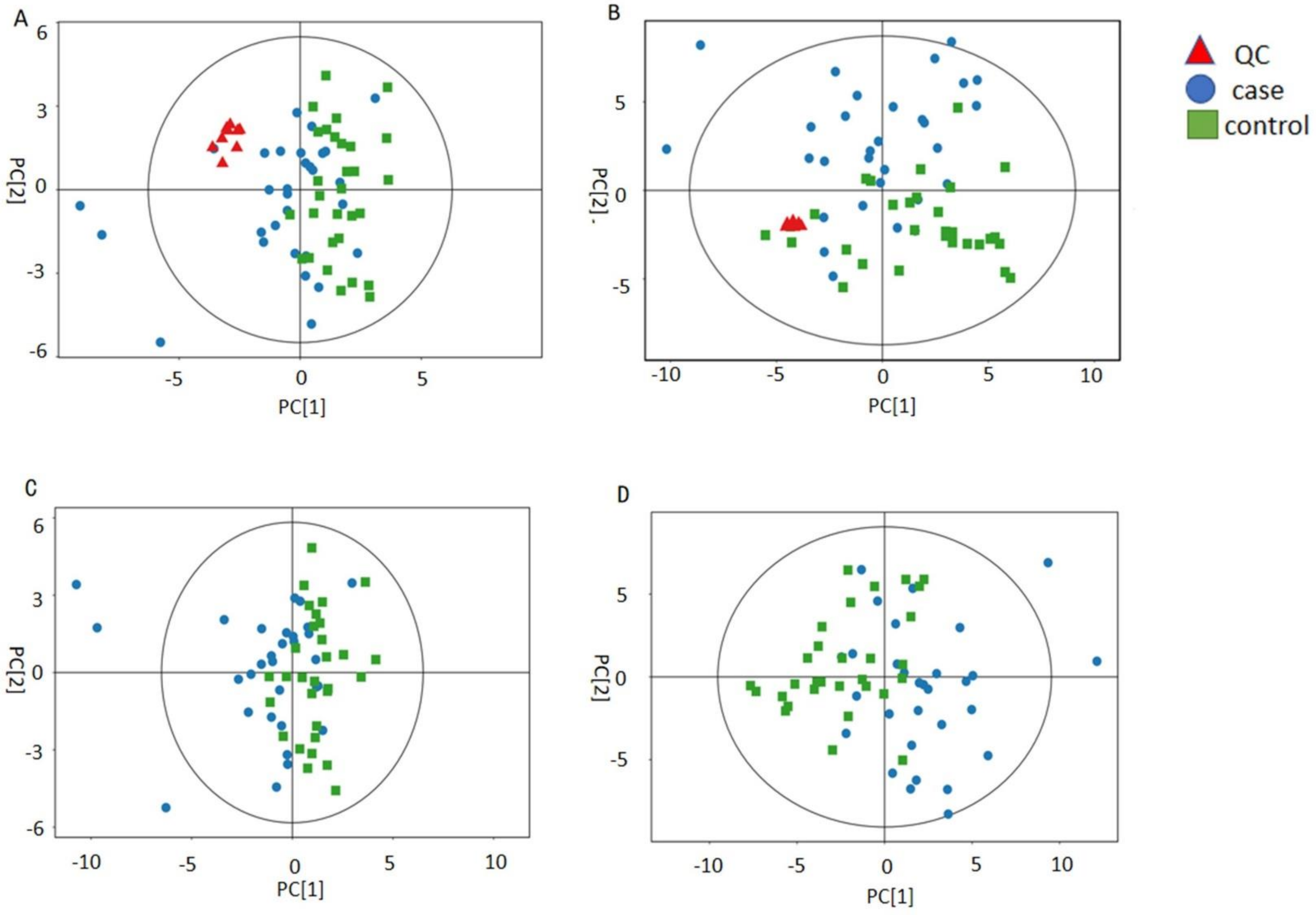

Supplemental Figure 2. PLS-DA score plot of the control and hyperthyroidism groups.

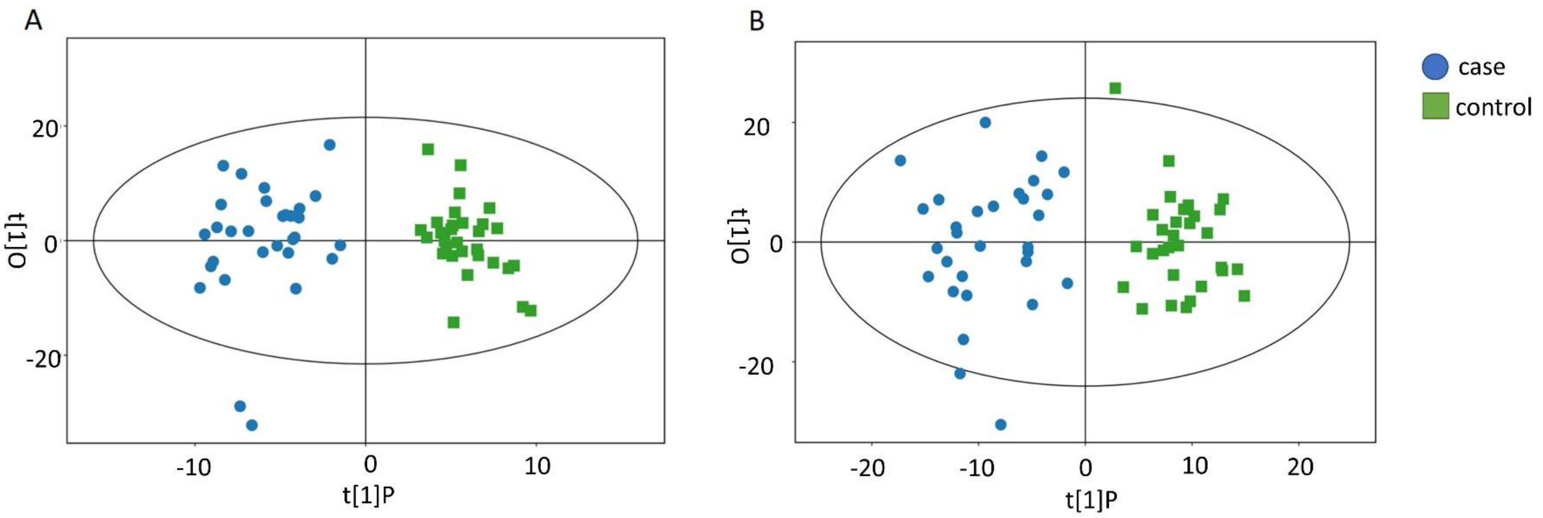

Supplement: Supplementary file 1 [file Image_1.pdf]
